# Supplementary material for: Associations between aversive learning processes and transdiagnostic psychiatric symptoms in a general population sample
Source: Nat Commun. 2020 Aug 21;11:4179. doi: 10.1038/s41467-020-17977-w (PMC7443146; doi:10.1038/s41467-020-17977-w)
Supplement: Supplementary file 3 — Reporting Summary [file 41467_2020_17977_MOESM3_ESM.pdf]

Corresponding author(s): *Double-blind peer review submissions: write DBPR and your manuscript number here instead of author names.*

Last updated by author(s): *YYYY-MM-DD*

# Reporting Summary

Nature Research wishes to improve the reproducibility of the work that we publish. This form provides structure for consistency and transparency in reporting. For further information on Nature Research policies, see our Editorial Policies and the Editorial Policy Checklist.

## Statistics

For all statistical analyses, confirm that the following items are present in the figure legend, table legend, main text, or Methods section.

| n/a | Confirmed | |
|---|---|---|
| ☐ | ☒ | The exact sample size (*n*) for each experimental group/condition, given as a discrete number and unit of measurement |
| ☐ | ☒ | A statement on whether measurements were taken from distinct samples or whether the same sample was measured repeatedly |
| ☐ | ☒ | The statistical test(s) used AND whether they are one- or two-sided<br>*Only common tests should be described solely by name; describe more complex techniques in the Methods section.* |
| ☐ | ☒ | A description of all covariates tested |
| ☐ | ☒ | A description of any assumptions or corrections, such as tests of normality and adjustment for multiple comparisons |
| ☐ | ☒ | A full description of the statistical parameters including central tendency (e.g. means) or other basic estimates (e.g. regression coefficient) AND variation (e.g. standard deviation) or associated estimates of uncertainty (e.g. confidence intervals) |
| ☐ | ☒ | For null hypothesis testing, the test statistic (e.g. *F*, *t*, *r*) with confidence intervals, effect sizes, degrees of freedom and *P* value noted<br>*Give P values as exact values whenever suitable.* |
| ☐ | ☒ | For Bayesian analysis, information on the choice of priors and Markov chain Monte Carlo settings |
| ☐ | ☒ | For hierarchical and complex designs, identification of the appropriate level for tests and full reporting of outcomes |
| ☐ | ☒ | Estimates of effect sizes (e.g. Cohen's *d*, Pearson's *r*), indicating how they were calculated |

*Our web collection on statistics for biologists contains articles on many of the points above.*

## Software and code

Policy information about availability of computer code

| | |
|---|---|
| Data collection | Javascript, Phaser 3 |
| Data analysis | Python 2.7, PyMC3, Scikit-Learn |

For manuscripts utilizing custom algorithms or software that are central to the research but not yet described in published literature, software must be made available to editors and reviewers. We strongly encourage code deposition in a community repository (e.g. GitHub). See the Nature Research guidelines for submitting code & software for further information.

## Data

Policy information about availability of data

All manuscripts must include a data availability statement. This statement should provide the following information, where applicable:

- Accession codes, unique identifiers, or web links for publicly available datasets
- A list of figures that have associated raw data
- A description of any restrictions on data availability

All data supporting the findings of this study are available through the Open Science Framework at https://osf.io/b95w2/ with the data DOI 10.17605/OSF.IO/B95W2. A reporting summary for this article is available as a Supplementary Information File.

# Field-specific reporting

Please select the one below that is the best fit for your research. If you are not sure, read the appropriate sections before making your selection.

☐ Life sciences ☒ Behavioural & social sciences ☐ Ecological, evolutionary & environmental sciences

For a reference copy of the document with all sections, see nature.com/documents/nr-reporting-summary-flat.pdf

# Behavioural & social sciences study design

All studies must disclose on these points even when the disclosure is negative.

| | |
|---|---|
| Study description | This study was an experimental study of aversive learning conducted online using quantitative methods. We used an aversive learning task in conjunction with questionnaire measures of psychopathology. |
| Research sample | The sample was recruited online through Prolific. All subjects were aged 18-65, and the sample had a mean age of 30.27 and were 53% female. We did not seek to recruit a representative sample, but sought to recruit a large sample from the general population. Thus, this was a sample of subjects who participate in studies on Prolific. The rationale for this sample was A) convenience, in that we wished to conduct the research online, and B) representativeness, in that online samples provide more representative subjects than lab-based studies using undergraduates. |
| Sampling strategy | We used convenience sample with a precision-based stopping rule to determine our sample size, stopping at the point at which either the 95% highest posterior density interval (HPDI) for all effects in our regression model reached 0.15 (checking with each 50 subjects recruited) or we had recruited 400 subjects. The precision target was not reached, and so we stopped at 400 subjects. |
| Data collection | Subjects completed the experiment remotely with no experimenter present. Data was collected automatically via a web server, hosted on www.pavlovia.org. This recorded data through Javascript scripts. |
| Timing | Data were collected between 6/12/18 and 9/12/18 |
| Data exclusions | Exclusion criteria were pre-determined. We excluded subjects who did not provide complete data, and subjects who did not respond above a given threshold. However no subjects met either of these criteria and so no subjects were excluded. |
| Non-participation | No subjects dropped out or declined participation |
| Randomization | Subjects were not randomly allocated to conditions, and we controlled for potential confounds in all of our analyses (age, sex, and motivation on the task) by including them as covariates in regression analyses. |

# Reporting for specific materials, systems and methods

We require information from authors about some types of materials, experimental systems and methods used in many studies. Here, indicate whether each material, system or method listed is relevant to your study. If you are not sure if a list item applies to your research, read the appropriate section before selecting a response.

## Materials & experimental systems

| n/a | Involved in the study | |
|---|---|---|
| ☒ | ☐ | Antibodies |
| ☒ | ☐ | Eukaryotic cell lines |
| ☒ | ☐ | Palaeontology and archaeology |
| ☒ | ☐ | Animals and other organisms |
| ☐ | ☒ | Human research participants |
| ☒ | ☐ | Clinical data |
| ☒ | ☐ | Dual use research of concern |

## Methods

| n/a | Involved in the study | |
|---|---|---|
| ☒ | ☐ | ChIP-seq |
| ☒ | ☐ | Flow cytometry |
| ☒ | ☐ | MRI-based neuroimaging |

## Human research participants

Policy information about studies involving human research participants

| | |
|---|---|
| Population characteristics | See above |
| Recruitment | Subjects were recruited online through Prolific. There may be self-selection bias due to the fact that subjects completing studies online are generally relatively young and familiar with online services such as Prolific. However we do not expect this to have a significant impact on our results, and this approach arguably provides a more representative sample than traditional lab-based experiments on university students. One significant issue is the application of our results to clinical |

samples. We did not seek to recruit a clinical sample, instead looking at variation in traits related to mental health problems in a general population sample, and so we cannot be certain our results will generalise to those with diagnosed disorders.

| | |
|---|---|
| Ethics oversight | The protocol was approved by the University College London research ethics committee |

Note that full information on the approval of the study protocol must also be provided in the manuscript.
