# Supplementary material for: Associations between aversive learning processes and transdiagnostic psychiatric symptoms in a general population sample
Source: Nat Commun. 2020 Aug 21;11:4179. doi: 10.1038/s41467-020-17977-w (PMC7443146; doi:10.1038/s41467-020-17977-w)
Supplement: Supplementary file 2 — Peer Review File [file 41467_2020_17977_MOESM2_ESM.pdf]

Editorial Note: This manuscript has been previously reviewed at another journal that is not operating a transparent peer review scheme. This document only contains reviewer comments for versions considered at *Nature Communications*.

***REVIEWERS' COMMENTS:

Reviewer #2 (Remarks to the Author):

After transfer to Nature Communications I have now read the responses carefully. The authors have responded in detail to my concerns. The basic issues have been addressed and the more fundamental issues await further experiments (e.g. measurement the test/re-test characteristics of the paradigm). The insights provided by this approach are useful and the paradigm is interesting as it provides a probe to measure aversive learning. I appreciate the authors further highlighting some of the limitations of the current design.
